# Ultrasonographic study of fetal facial profile markers during the first trimester

**DOI:** 10.1186/s12884-021-03813-6

**Published:** 2021-04-24

**Authors:** Chunya Ji, Xiaoli Jiang, Linliang Yin, Xuedong Deng, Zhong Yang, Qi Pan, Jun Zhang, Qing Liang

**Affiliations:** Center for Medical Ultrasound, The Affiliated Suzhou Hospital of Nanjing Medical University, Suzhou Municipal Hospital, No. 26 Daoqian Street, 215002 Suzhou, Jiangsu China

**Keywords:** First trimester, Fetus, Facial profile, Marker, Ultrasonography

## Abstract

**Background:**

To establish reference ranges of fetal facial profile markers and study their correlations with crown-rump length (CRL) during the first trimester (11 ~ 13^+ 6^ weeks’ gestation) in a Chinese population.

**Methods:**

Ultrasonographic images of measuring fetal nuchal translucency (NT) were retrospectively selected randomly in normal fetuses whose parents were both Chinese. The facial markers included inferior facial angle (IFA), maxilla-nasion-mandible (MNM) angle, facial maxillary angle (FMA) and profile line (PL) distance. These markers were measured through ViewPoint 6 software by two experienced sonographers.

**Results:**

Three hundred and eighty fetuses were selected. The ICCs (95 % CI) of intra-operator 1 reproducibility of IFA, MNM angle, FMA, PL distance were 0.944 (0.886 ~ 0.973), 0.804 (0.629 ~ 0.902), 0.834 (0.68 ~ 0.918) and 0.935 (0.868 ~ 0.969), respectively. The ICCs (95 % CI) of intra-operator 2 reproducibility of IFA, MNM angle, FMA, PL distance were 0.931 (0.857 ~ 0.967), 0.809 (0.637 ~ 0.904), 0.786 (0.600 ~ 0.892) and 0.906 (0.813 ~ 0.954), respectively. The ICCs (95 % CI) of inter-operator reproducibility of IFA, MNM angle, FMA, PL distance were 0.885 (0.663 ~ 0.953), 0.829 (0.672 ~ 0.915), 0.77 (0.511 ~ 0.891) and 0.844 (0.68 ~ 0.925), respectively. The average ± SD of IFA, MNM angle, FMA and PL distance were 80.2°±7.25°, 4.17°±1.19°, 75.36°±5.31°, 2.78 ± 0.54 mm, respectively. IFA and PL distance significantly decreased with CRL, while MNM angle and FMA significantly increased with CRL.

**Conclusions:**

It was feasible to measure fetal facial markers during the first trimester. In Chinese population, the reference ranges of IFA, MNM angle, FMA and PL distance were 80.2°±7.25°, 4.17°±1.19°, 75.36°±5.31°, 2.78 ± 0.54 mm, respectively, and the measurements were found to correlate with CRL.

## Background

Fetal facial malformations mainly include cleft lip and palate (CLP), micrognathia, maxillary dysplasia, and absence of nasal bone, which are closely related to some chromosomal abnormalities or genetic syndrome [1, 2]. With the rapid development of ultrasound technology and the continuous accumulation of sonographers’ experience in recent years, the majority of CLP can be diagnosed mainly during the second and third trimester. Severe micrognathia can be subjectively judged based on the shape of fetal facial profile and also be assessed by measuring the mandible length [3]. However, if fetal facial malformations can be diagnosed during the first trimester (11 ~ 13^+ 6^ weeks’ gestation), healthcare providers and parents will have enough time to evaluate fetal prognosis, such as performing chorionic villus sampling (CVS) or early anatomic survey, which is of great clinical importance. Actually most facial structures of the fetus have been differentiated during the first trimester [4], so it is feasible to evaluate fetal facial structure during the first trimester [5]. The guideline issued in 2013 by International Society of Ultrasound in Obstetrics and Gynecology (ISUOG) [6] pointed out that it was crucial to observe the fetal facial profile. However, prenatal diagnosis of fetal facial abnormalities is still challenging in the first trimester around the world, and a series of simple, reliable and reproducible objective parameters are still lacking. In this study, fetal facial markers including inferior facial angle (IFA), maxilla-nasion-mandible (MNM) angle, facial maxillary angle (FMA) and profile line (PL) distance, were measured in fetal facial mid-sagittal section during the first trimester. The aim of the present study was to establish the reference range for each marker in Chinese population and analyze their correlation with CRL during the first trimester.

## Methods

### Study subjects

 Images of the first trimester ultrasound screening peformed in the Affiliated Suzhou Hospital of Nanjing Medical University between August 2017 and July 2019, which best met inclusion criteria and have high recognizable structure, were retrospectively selected. The pregnancy outcome was followed-up by the Suzhou Maternal-children health care system.

The inclusion criteria were as follows: (1) both parents of the fetus were Chinese; (2) pregnancies with significant maternal complications were excluded; (3) singleton pregnancy; (4) fetuses with normal ultrasound findings and normal follow-up outcomes; (5) the selected two-dimensional ultrasound (2D-US) images were the standard mid-sagittal section for measuring nuchal translucency (NT) thickness, which met the standardized protocol at 11 ~ 13^+6^ weeks’ gestation of the Fetal Medicine Foundation (FMF). The forehead, nasal bone, palate, mandible, upper lip, lower lip and other structures should be clearly displayed in this section.

 The study was approved by the Ethics Committee of Suzhou Municipal Hospital.

### Equipment and software

A Philips Affiniti70 and a GE Voluson E10 four-dimensional (4D) color ultrasound machines were utilized in this study; each was equipped with a convex probe of C9-2 and C5-1, with the frequency of 2 ~ 9 MHz and 1 ~ 5 MHz, respectively. The images obtained by transabdominal ultrasound examination were imported into the ultrasound workstation software, ViewPoint 6 in DICOM (Digital Imaging and Communications in Medicine) format. In addition, fetal facial markers were measured through ViewPoint 6.

### Definition of the markers

**Fig. 1:**
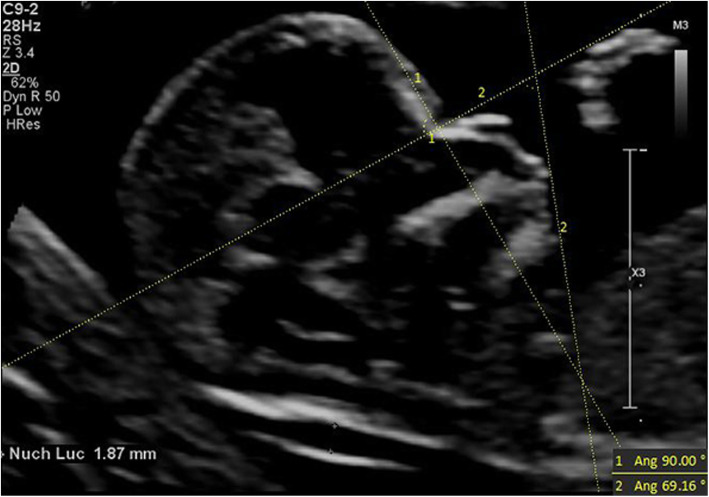
The measurement of IFA (69.16°); 13w2d, normal Chinese fetus

**Fig. 2:**
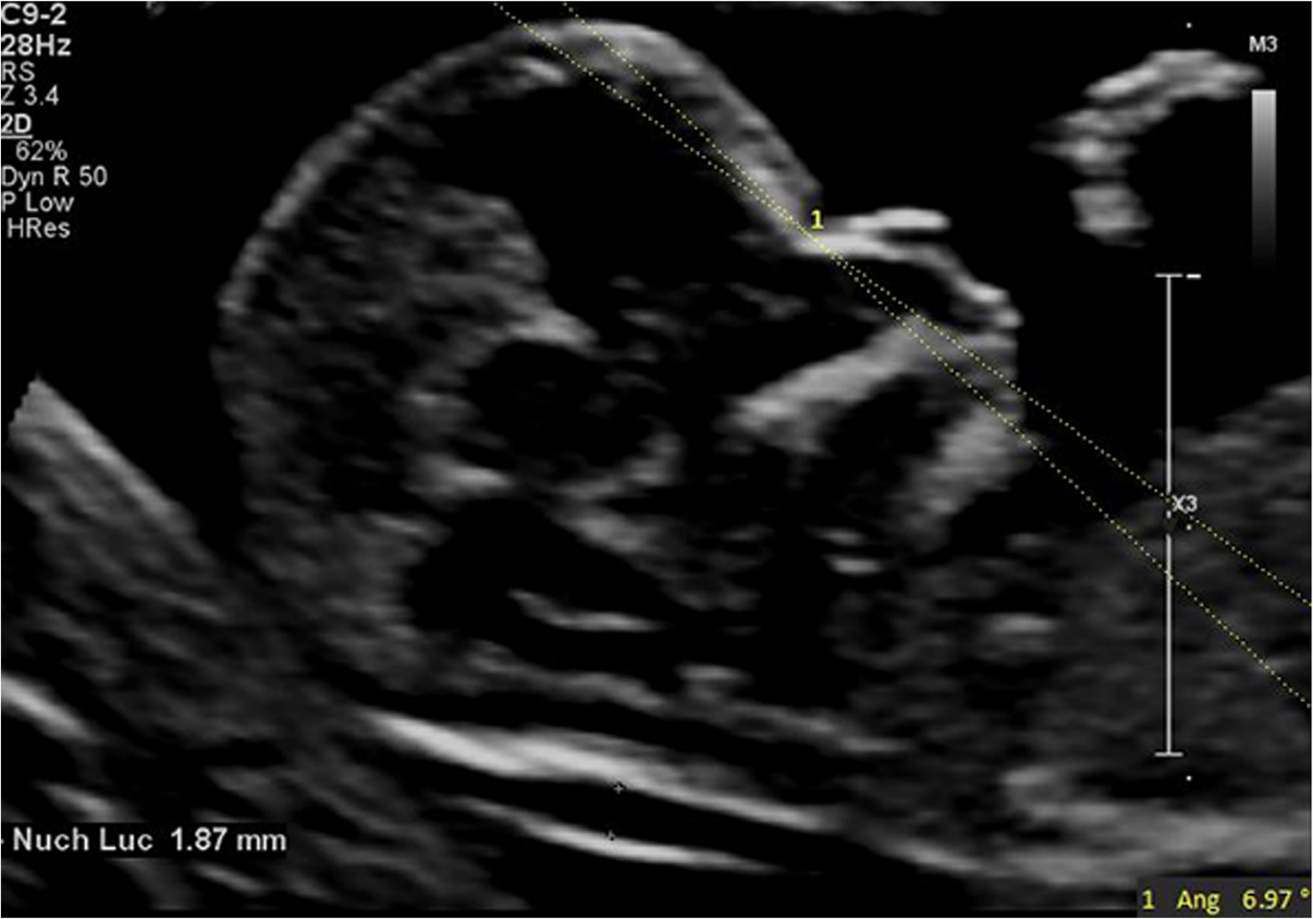
The measurement of MNM angle (6.97°); 13w2d, normal Chinese fetus

**Fig. 3:**
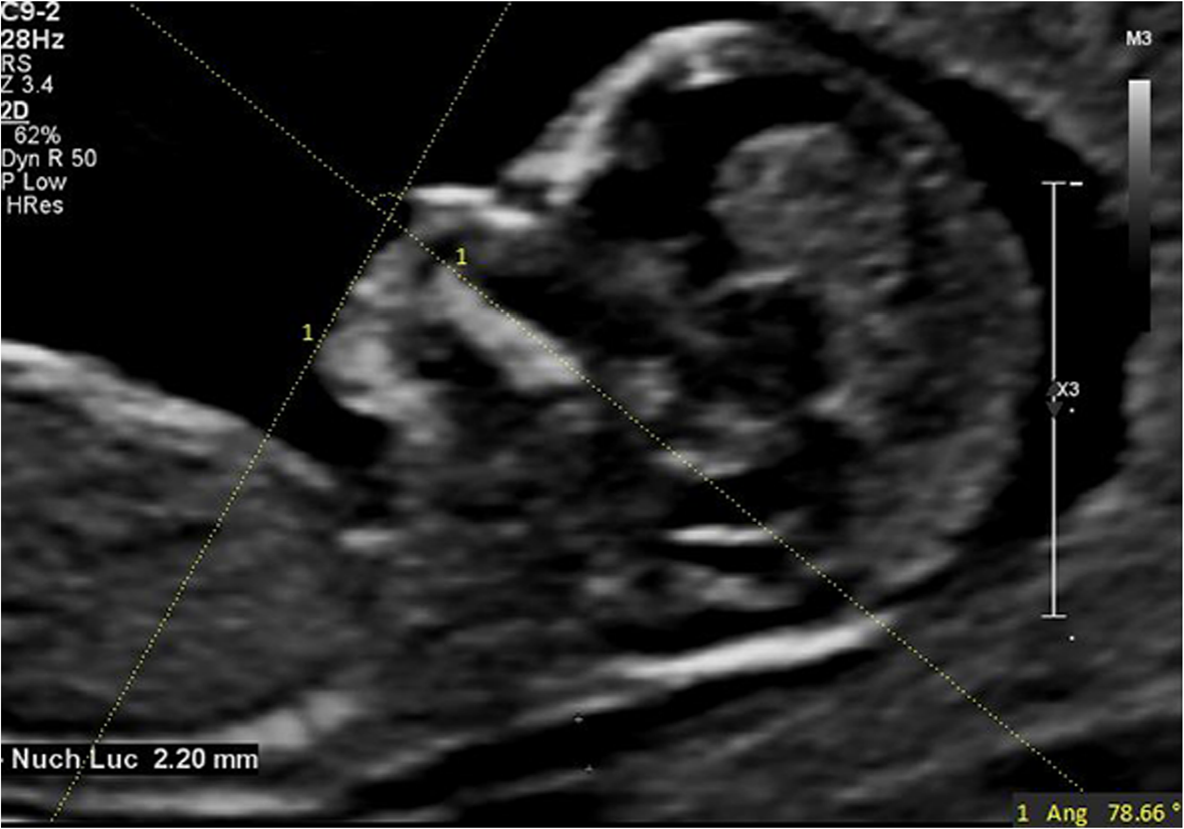
The measurement of FMA (78.66°); 13w6d, normal Chinese fetus

**Fig. 4:**
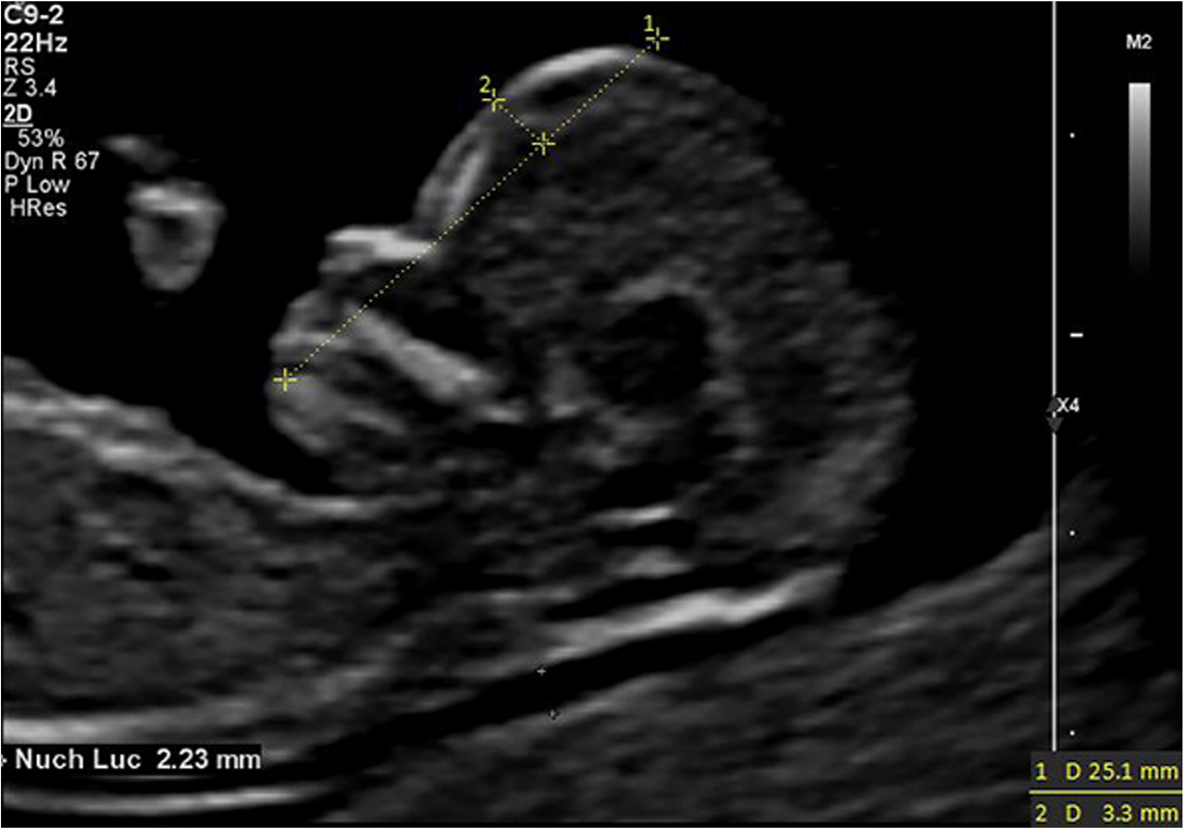
The measurement of PL distance (3.3mm) 12w4d, normal Chinese fetus

IFA [7] was defined as the angle between the line orthogonal to the vertical part of the forehead at the level of the synostosis of the nasal bone and the line joining the tip of the mentum to the most anterior point of the more protruding lip (Fig. 1). MNM angle [8] was defined as the angle between maxilla-nasion line and mandible-nasion line in the mid-sagittal section (Fig. 2), and the nasion [9] was defined as the most anterior point at the intersection of the frontal and nasal bone. FMA [10] was the angle between the line overlying the maxilla and the line across mentum tip and upper lip (Fig. 3). The FPL [9] was defined as the line that passed through the middle point of the anterior border of the mandible and the nasion. PL distance [9] was the perpendicular distance from the facial profile line (FPL) to the outer border of the forehead (Fig. 4).

### Measurement of the markers

Fetal facial markers (IFA, MNM angle, FMA, PL distance) were measured through ViewPoint 6 software by two experienced sonographers, who had obtained the FMF certification for NT scan. The average value of each marker was taken after three measurements. Generally speaking, it takes every 1.5-2 min to measure each of the four markers once, and every 5-6 min for three times.

### Statistical analysis

The analysis was performed by SPSS21.0 (Chicago, IL, USA) and Graphpad Prism8.0. The intraclass correlation coefficient (ICC) and Bland-Altman analysis [11] were used to assess intra-operator and inter-operator reproducibility. Bland-Altman mean and 95 % limits of agreement (LOA) were constructed. The reference range of the data with a Gaussian distribution was expressed by mean ± 1.96standard deviation (SD). The reference range of the data without a Gaussian distribution was expressed by Median (Inter-Quartile Range). Pearson correlation analysis and univariate regression analysis investigated the correlation between fetal facial markers and CRL.

## Results

**Fig. 5:**
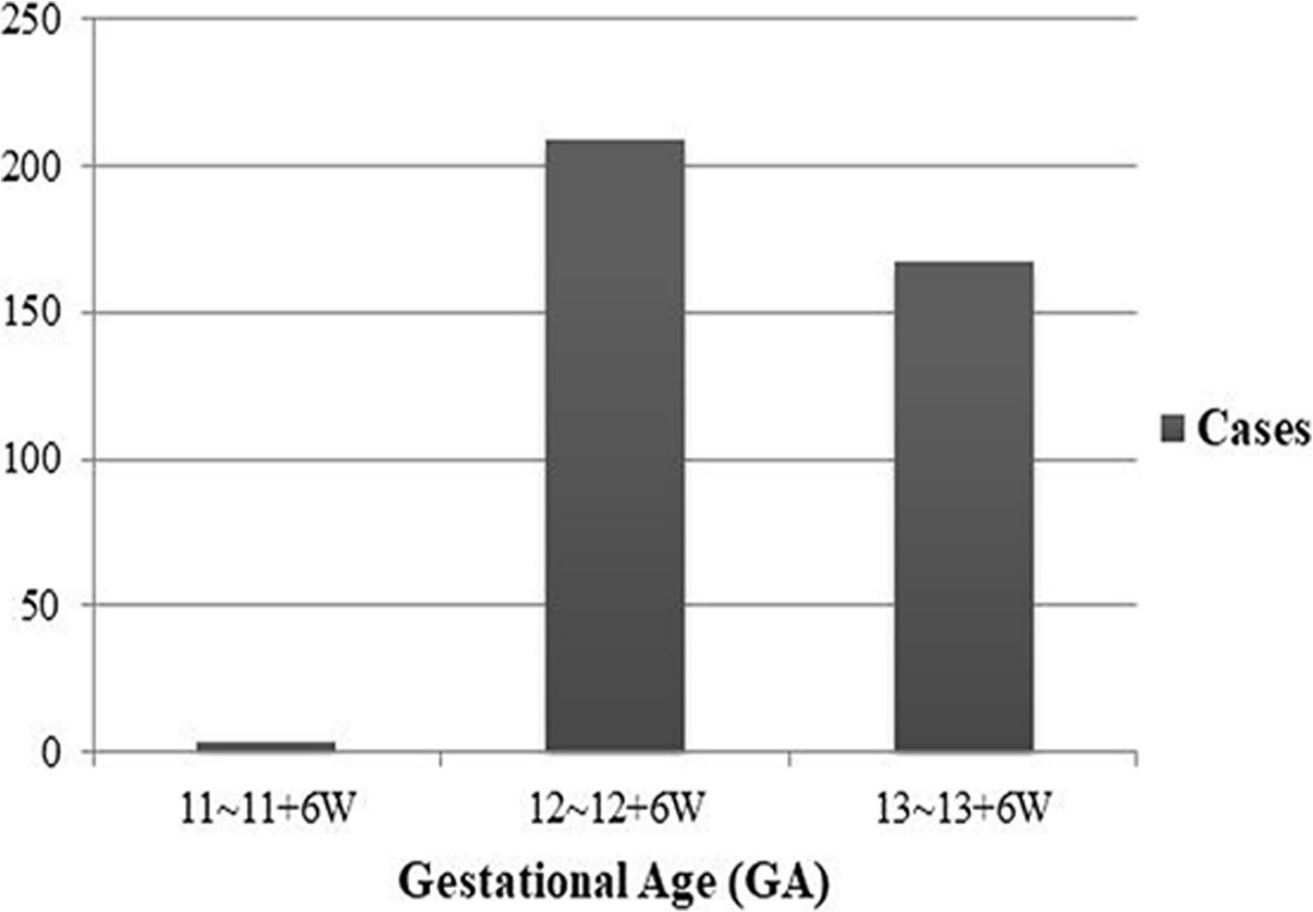
The distribution of the cases of fetuses in each gestational age

Among 3520 fetuses who underwent the first trimester ultrasound screening, 380 were selected because they met the inclusion criteria. The successful rate of measuring all four facial markers is almost 100 %. The maternal age was 28 (4) years old. The CRL was 66 (10) mm, and the NT thickness was 1.80 (0.5) mm. The distribution of fetal cases in each gestational week is shown in Fig. 5.

### Evaluation of intra‐operator and inter‐operator agreement

Thirty ultrasonographic images in fetal facial mid-sagittal section were randomly selected from 380 fetuses that met the inclusion criteria to evaluate the reproducibility and feasibility of the measurement. The ICCs (95 % CI) of intra-operator 1 reproducibility of IFA, MNM angle, FMA, PL distance were 0.944 (0.886 ~ 0.973), 0.804 (0.629 ~ 0.902), 0.834 (0.68 ~ 0.918) and 0.935 (0.868 ~ 0.969), respectively. The ICCs (95 % CI) of intra-operator 2 reproducibility of IFA, MNM angle, FMA, PL distance were 0.931 (0.857 ~ 0.967), 0.809 (0.637 ~ 0.904), 0.786 (0.600 ~ 0.892) and 0.906 (0.813 ~ 0.954), respectively. The ICCs (95 % CI) of inter-operator reproducibility of IFA, MNM angle, FMA, PL distance were 0.885 (0.663 ~ 0.953), 0.829 (0.672 ~ 0.915), 0.77 (0.511 ~ 0.891) and 0.844 (0.68 ~ 0.925), respectively.

**Fig. 6:**
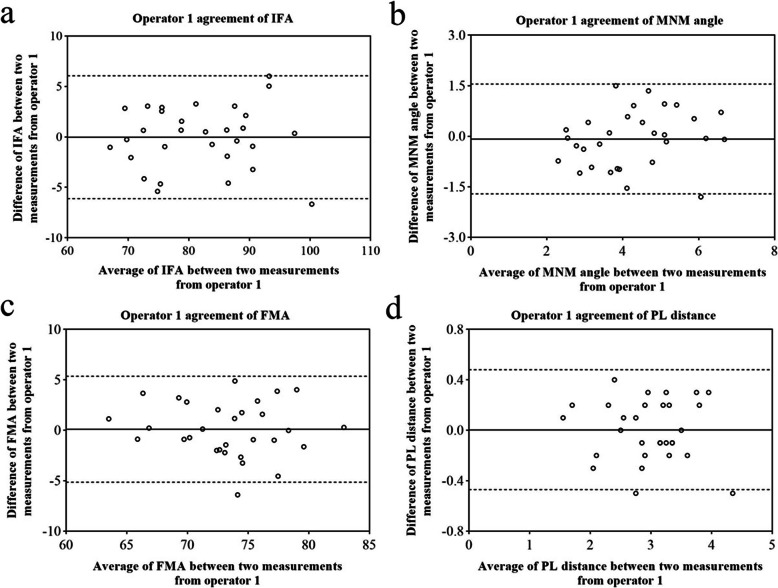
Operator 1 agreement in the measurement of IFA (**a**), MNM angle (**b**), FMA (**c**) and PL distance (**d**) solid line: the mean of the difference of the paired measurements; dotted line: 95 %LOA of the difference

**Fig. 7:**
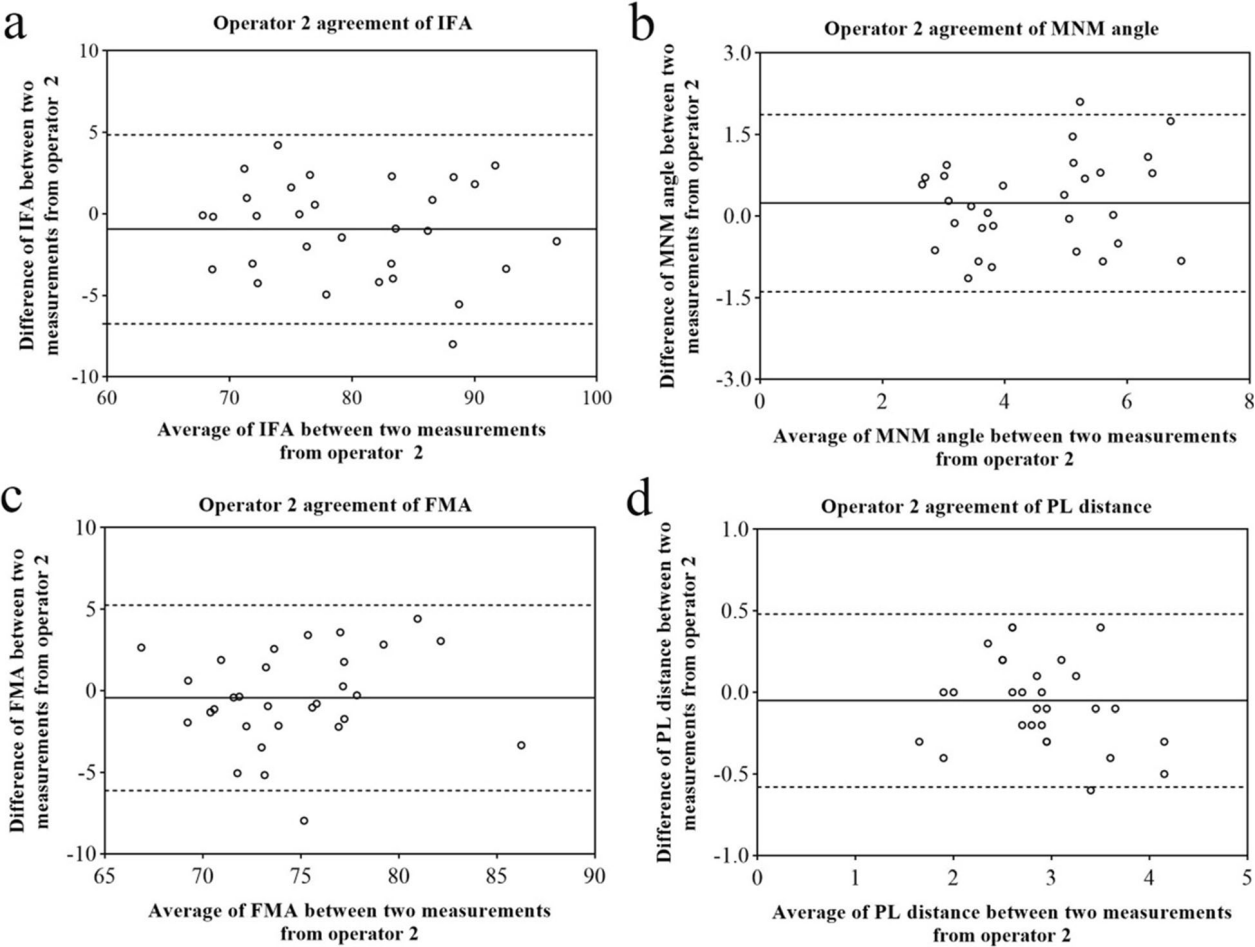
Operator 2 agreement in the measurement of IFA (**a**), MNM angle (**b**), FMA (**c**) and PL distance (**d**) solid line: the mean of the difference of the paired measurements; dotted line: 95 %LOA of the difference

**Fig. 8:**
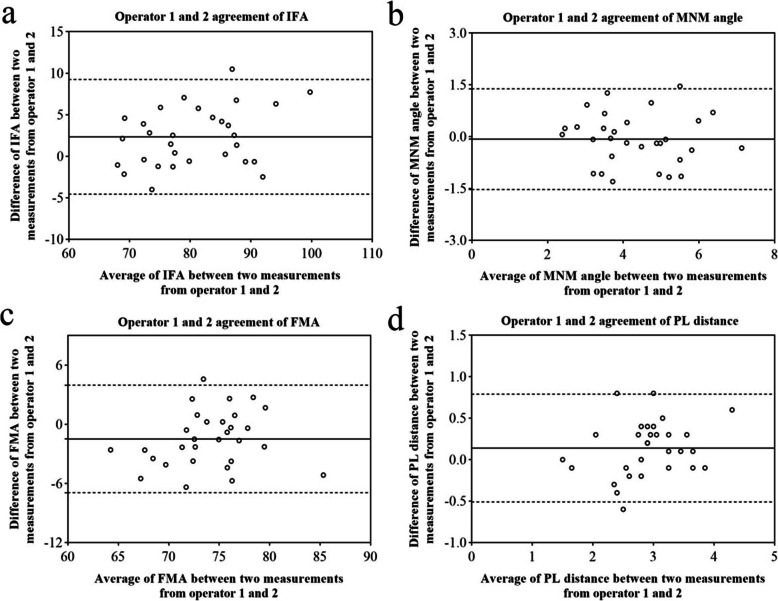
Operator 1 and 2 agreement in the measurement of IFA (**a**), MNM angle (**b**), FMA (**c**) and PL distance (**d**) solid line: the mean of the difference of the paired measurements; dotted line: 95 %LOA of the difference

Table 1; Figs. 6a-d, 7a-d and 8a-d showed the Bland-Altman analysis evaluating intra-operator and inter-operator the agreement of measurement of IFA, MNM angle, FMA and PL distance. The reproducibility of these markers for intra-operator and inter-operator was good.

**Table 1:** Intra-operator and inter-operator agreement of IFA, MNM angle, FMA and PL distance

	operator 1		operator 2		operator 1 and 2	
	mean	95 %LoA	mean	95 %LoA	mean	95 %LoA
IFA	-0.02	-6.10 ~ 6.05	-0.95	-6.75 ~ 4.85	2.34	-4.57 ~ 9.25
MNM angle	-0.08	-1.71 ~ 1.55	0.24	-1.39 ~ 1.87	-0.068	-1.53 ~ 1.39
FMA	0.11	-5.14 ~ 5.36	-0.43	-6.11 ~ 5.26	-1.49	-6.94 ~ 3.97
PL distance	0.003	-0.47 ~ 0.48	-0.05	-0.58 ~ 0.48	0.14	-0.51 ~ 0.79

### Correlation between fetal facial markers and CRL

The measurement range of IFA was 55.9°~107.89° (80.2°±7.25°). IFA had significant negative correlation to CRL during the first trimester (IFA = 127.601–0.707*CRL,r=-0.598, *p* < 0.001). IFA was Gaussian distributed, and its reference range was 65.99°~94.41° (mean ± 1.96SD).

The measurement range of MNM angle was 1.66°~9.21° (4.17°±1.19°). The MNM angle had significant positive correlation to CRL during the first trimester (MNM angle=-4.112 + 0.123*CRL,r = 0.547, *p* < 0.001). The MNM angle was Gaussian distributed, and the reference range of the MNM angle was 1.84°~6.50° (mean ± 1.96SD).

The measurement range of FMA was 56.29°~89.59° (75.36°±5.31°). FMA depended significantly on CRL (FMA = 55.683 + 0.293*CRL,r = 0.339, *p* < 0.001). FMA was Gaussian distributed, and the reference range was 64.95°~85.77° (mean ± 1.96SD).

The measurement range of PL distance was 1.53 ~ 4.37mm (2.78 ± 0.54mm). The PL distance decreased with CRL (PL distance = 5.136 − 0.035*CRL,r=-0.399, *p* < 0.001). The PL distance was Gaussian distributed, and its reference range was 1.72 ~ 3.84mm (mean ± 1.96SD).

## Discussion

Trisomy 21, also known as Down Syndrome (DS), is the most common chromosomal abnormality and is accompanied by different degrees of mid-facial hypoplasia and skin edema [12, 13]. Compared to euploid fetuses, DS fetuses have typical facial features such as hypoplastic or absent nasal bones, thickened prenasal skin, shortening and dorsal displacement of the maxilla, et al. [14]. Most fetuses with trisomy 18, the second most common chromosomal abnormality, have micrognathia [15] and CLP. The diagnosis of micrognathia is mainly subjective during the second and third trimester. In the meanwhile, CLP is the most common facial malformation. Although not fatal, it has great adverse impact on children and families involved. Moreover, 54 % of CLP may associated with other anomalies or genetic syndromes [16], affecting about 1 ~ 2/1000 live births [17].

With the rapid development of science and technology, NIPT (non-invasive prenatal test) has become the first choice for screening chromosomal aneuploidy due to its non-invasiveness, high sensitivity, and high specificity [18]. To some extent, the value of these facial markers in predicting aneuploidy was limited. NIPT has a high detection rate for DS, trisomy 18 and trisomy 13, but it cannot completely cover 23 pairs of chromosomes. The first trimester ultrasound screening could diagnosis some structural abnormalities such as CLP and micrognathia, and this advantage could not be replaced by NIPT. In this study, multiple facial markers reflected the relative position of the forehead, maxilla, and mandible were analyzed to establish their reference ranges, and these markers could further provide objective and quantitative criteria for the early detection of fetal facial anomalies and underlying genetic abnormalities.

The embryonic development of facial bones has its main characteristics. The maxilla and mandible begin to ossify from 8 weeks onward [19]. The maxilla is anatomically fused with the skull and grows forward with the development of brain tissue, while the mandible is connected to the skull through the temporomandibular joint. Therefore, the mandibular forward growth rate during the first trimester is slower than that of the maxilla [20]. From 20 weeks onward, the maxilla ossification has almost completed, and the developing fetal swallowing function accelerates the growth of mandible. After that, the position of facial bones is relatively constant, then fetal facial profile is basically formed [21].

In 2002, Rotten et al. [7] studied 371 normal fetuses and 12 fetuses with mandible anomalies and first introduced the IFA to detect micrognathia. They found that the mean IFA of normal fetuses was 65.5°±8.13° at 18 ~ 28 weeks’ gestation, and it was constant during pregnancy. Using 49.2° (average-2SD) as a cut-off, the IFA had a sensitivity of 1.0, a specificity of 0.989, a false positive rate of 0.011 to predict micrognathia. IFA could reflect the anterior and posterior position between the mandible and frontal bone to evaluate micrognathia, which was often associated with some genetic anomalies, such as Pierre-Robin syndrome, Stickler syndrome, trisomy 18 and trisomy 13 [1, 22, 23]. During the first trimester, we found that the mean IFA of normal fetuses was 80.2 (SD 7.25) °, and it decreased with CRL. This value was close to Tekesin et al. (76.5°±6.30°) [24] but larger than Rotten et al. [7]. The reason might be that the mandibular forward growth rate during the first trimester is slower than that of the forehead. While during the second trimester, the position of facial bones is relatively constant. Similar to our results, Orzechowski et al. [25] and Tekesin et al. [24] also reported the IFA decreased with CRL during the first trimester. According to their results, the abnormal IFA did not seem to be helpful for diagnosis of trisomy 21 [24, 25]. In our study, the reference range of IFA was 65.99°~94.41°. The possibility of micrognathia, which was relevant for the early detection of certain genetic syndromes, should be considered when IFA was less than 65.7° (average-2SD). However, the clinical significance of this supposed value needs to be confirmed by large abnormal sample from multi-centers.

The MNM angle could reflect the relative position of the maxilla and mandible, further to evaluate fetal facial profile. De Jong-Pleij et al. [8] reported that the mean MNM angle was 13.5° and independent of gestational age in the second and third trimester, making it a sensitive indicator for evaluating micrognathia and CLP. Vos et al. [26, 27] reported that the MNM angle had a definite implication for trisomy 21 and trisomy 18 during later stages of pregnancy. In our study, the mean MNM angle was 4.17 (SD 1.19) °, which increased with CRL. However, Ko et al. [28] found the MNM angle had a negative correlation with gestational age at 14 ~ 39 weeks. Lu et al. [10] pointed out that the MNM angle did not change at 16 ~ 36 weeks’ gestation. Recently, Sun et al. [18] demonstrated that the MNM angle was higher in euploid fetuses than trisomy 21 fetuses during the first trimester. Further studies are necessary to investigate the relationship between the MNM angle and gestational age in order to diagnose more fetal facial abnormalities or chromosomal abnormalities.

FMA can directly reflect the relative position of the maxilla and mandible to evaluate fetal facial profile. In order to avoid the influence of the curvature of the vomer, Lu et al. [10] used the surface of anterior half of the maxilla as a reference line. Their research showed that FMA was related to gestational age which increased with gestation slightly (1°~ 2°/week) from 16 weeks to 28 ~ 31 weeks and then decreased minimally. It might be consistent with the allometric growth relationship between different parts of fetal face. We found that FMA increased with CRL, with the reference range of 64.95°~85.77°. Lu et al. [10] reported that the cut-off of FMA in detecting micrognathia was 66° (average-2SD at 16 weeks), with the detection rate of 100 % and false positive rate of 2.5 %. The value was similar to our study (64.95°). A large prospective cohort is needed to determine the diagnostic accuracy of FMA for micrognathia during the first trimester.

De Jong-Pleij et al. [29] showed that the mean PL distance at 27 ~ 36 weeks’ gestation was 2.8 (range 2.1 ~ 3.6) mm, and 4 mm could be used as the upper limit of the normal for judging frontal bossing. The PL distance was the first objective quantitative indicator to assess frontal bossing, which was affected by the position of the mandible, nasion and frontal bone. In our study, the mean PL distance was 2.78 ± 0.54mm, and it decreased with CRL, which was consistent with Bakker et al. [9]. This might be caused by forward movement of the maxilla and decrease in convexity of the forehead during the first trimester [20]. Bakker et al. [9] also pointed out that the PL distance was not the best ultrasound marker for aneuploidies.

There were some limitations in our study. Firstly, it is a retrospective study, and the static image selected from the first trimester ultrasound screening was to measure the NT thickness rather than observe facial abnormalities. Secondly, the same stored ultrasound image was used by the two operators to assess the inter-operator reproducibility, and thus accounted at least partly for the high ICC. Thirdly, facial abnormalities with or without aneuploidies were not included. In addition, all parameters were measured on 2D images, without the use of 3D reconstructed techniques. Some research [7, 14] showed that 3D technique could better obtain the true mid-sagittal section, but it took a long time. On the other hand, 2D ultrasound was the basis of 3D ultrasound. 2D measurements were reported to be the same reliable and accurate as 3D measurements in the measurement of facial marker [30, 31].

## Conclusions

Intra-operator and inter-operator reproducibility for facial profile markers (IFA, MNM angle, FMA, PL distance) were good during the first trimester (11 ~ 13^+ 6^ weeks’ gestation). It’s feasible to measure these markers during the first trimester. The reference range of each marker was obtained through large sample data, and these markers were significantly related to CRL. If the measurement is not in this range, micrognathia and other facial abnormalities should be highly suspected. On this basis, further studies will be carried out to investigative the detection rate and false positive rate of these markers for facial abnormalities with or without aneuploidies. However, their clinical application shall be cautious. A large multi-center prospective cohort and enough positive cases are required.

## Data Availability

The datasets and code used and analyzed during the current study are available from the corresponding author on reasonable request.

## Abbreviations

CLP: Cleft lip and palate
CRL: Crown-rump length
CVS: Chorionic villus sampling
DICOM: Digital Imaging and Communications in Medicine
DS: Down Syndrome
FMA: Facial maxillary angle
FMF: Fetal Medicine Foundation
FPL: facial profile line
ICC: Intraclass correlation coefficient
IFA: Inferior facial angle
ISUOG: International Society of Ultrasound in Obstetrics and Gynecology
LOA: Limits of agreement
MNM: Maxilla-nasion-mandible angle
NIPT: Non-invasive prenatal test
NT: Nuchal translucency
PL: Profile line distance
SD: Standard deviation
2D-US: Two-dimensional ultrasound
4D: Four-dimensional
